# Genetic evidence from mitochondrial DNA corroborates the origin of Tibetan chickens

**DOI:** 10.1371/journal.pone.0172945

**Published:** 2017-02-27

**Authors:** Long Zhang, Pu Zhang, Qingqing Li, Uma Gaur, Yiping Liu, Qing Zhu, Xiaoling Zhao, Yan Wang, Huadong Yin, Yaodong Hu, Aiping Liu, Diyan Li

**Affiliations:** 1 Farm Animal Genetic Resources Exploration and Innovation Key Laboratory of Sichuan Province, Sichuan Agricultural University, Chengdu, China; 2 Life Science College, Southwest Forestry University, Kunming, China; 3 College of Food Science, Sichuan Agricultural University, Ya'an, China; Sichuan University, CHINA

## Abstract

Chicken is the most common poultry species and is important to human societies. Tibetan chicken (*Gallus gallus domesticus*) is a breed endemic to China that is distributed mainly on the Qinghai-Tibet Plateau. However, its origin has not been well characterized. In the present study, we sequenced partial mitochondrial DNA (mtDNA) control region of 239 and 283 samples from Tibetan and Sichuan indigenous chickens, respectively. Incorporating 1091 published sequences, we constructed the matrilineal genealogy of Tibetan chickens to further document their domestication history. We found that the genetic structure of the mtDNA haplotypes of Tibetan chickens are dominated by seven major haplogroups (A-G). In addition, phylogenetic and network analyses showed that Tibetan chickens are not distinguishable from the indigenous chickens in surrounding areas. Furthermore, some clades of Tibetan chickens may have originated from game fowls. In summary, our results collectively indicated that Tibetan chickens may have diverged from indigenous chickens in the adjacent regions and hybridized with various chickens.

## Introduction

As one of the most extensively distributed domesticated animals, chicken not only provides humans with a stable source of protein, but also serves important roles in numerous aspects of human society [1–3]. Tibetan chicken is an indigenous breed at high altitudes and is well known for its adaptability to survive in hypoxic conditions [4]. For example, the hatchability of Tibetan chicken eggs incubated in high attitude is greater than 70%, whereas hatchability is less than 40% for low altitude breeds [5, 6]. The hypoxia adaptation is achieved in Tibetan chickens via an increased red blood cell (RBC) count and blood oxygen affinity, together with a decreased mean cell volume and reduced susceptibility to hypocapnia [7]. To date, numerous genetic association studies demonstrated the hypoxia adaptability of Tibetan chickens living at high altitudes [8–11], yet the origin and evolution of this breed endemic to China is unknown.

Mitochondrial DNA (mtDNA) is inherited maternally and is often employed in population genetic analyses due to its high copy number, haploid nature and absence of/rare recombination events [12, 13]. The relative high mutation rate makes it suitable in studying domestic animals with recent timescale [14], numerous studies have focused on reconstructing the matrilineal history of domestic chickens by studying the control region of mtDNA. In initial researches, Fumihito et al [15, 16] have suggested a monophyletic origin of domestic chickens; these authors propose that the domestic chickens’ primary wild ancestor is a group of red jungle fowls found in the forests of Southeast Asia and India, which was spread to other parts of the world when people domesticated chickens, resulting in many chicken breeds. In contrast, with the increasing sample size, other authors have provided evidence of numerous and independent domestication events. Liu et al [17] suggested the involvement of multiple matrilines in domestication events in southern China, South Asia and Southeast Asia. Meanwhile, Kanginakudru et al [18] found evidences of domestication in Indian birds from *G*. *g*. *spadiceus*, *G*. *g*. *gallus* and *G*. *g*. *murghi*, demonstrating multiple domestication pathways of Indian and other domestic chickens. Recent studies involving the analysis of chicken mtDNA sequences from ancient sequences indicated some potential time, region, and pattern of chicken domestication in several regions [19–22], thus suggesting the key role of control region sequences in determining the history of chickens domestications.

In the present study, we used mtDNA partial control region sequences to investigate (i) the genetic diversity of Tibetan and indigenous fowls; (ii) the evolutionary status of Tibetan chickens as well as the surrounding indigenous populations; and (iii) the potential origin of Tibetan chickens. Our findings will increase the understanding about the matrilineal origin of Tibetan chickens in China.

## Materials and methods

### Sampling and DNA extraction

**Fig 1:**
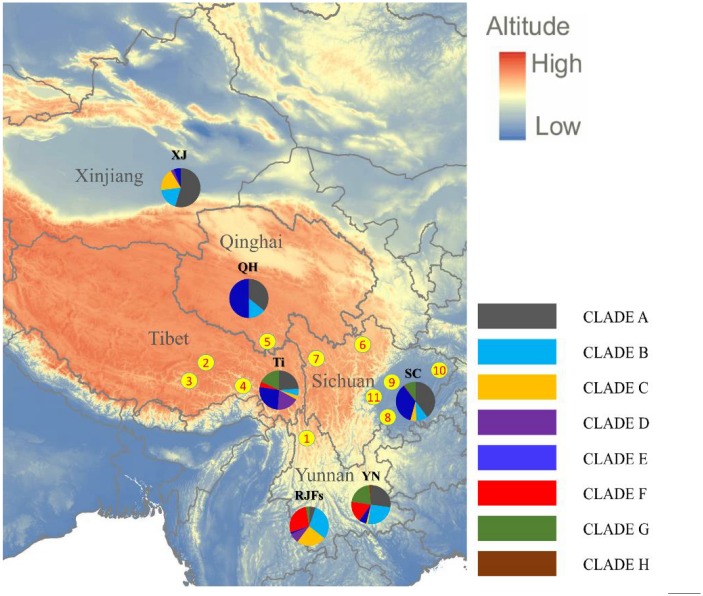
Map of sampling localities and distributions of the domestic haplogroups in each group. Note: Codes 1–7 represent the sampling localities of Tibetan chicken populations from Diqing (27°49′ N, 99°42′ E), Linzhi (3100 m; 29°39′ N, 94°21′ E), Lhasa (3650 m; 29°38′ N, 91°06′ E), Shannan (3700 m; 29°13 N, 91°44′ E), Haiyan (3260 m; 36°53′ N, 100°59′ E), Aba (3300 m; 32°54′ N, 101°42′ E) and Ganzi (3390 m; (31°37′ N, 99°59′ E), respectively. Codes 8–12 represent the sampling localities of Sichuan lowland chicken populations from Leshan (400 m; 29°33′ N, 103°45′ E), Chengdu (540 m; 30°34′ N, 104°03′ E), Dazhou (800 m; 30°12′ N, 107°27′ E), Ya’an (900 m; 30°01′ N, 103°02′ E) and Panzhihua (1400 m; 26°35′ N, 101°43′ E), respectively. Ti: Tibetan chickens; SC: Sichuan indigenous chickens; YN: Yunnan indigenous chickens; XJ: Xinjiang indigenous chickens; QH: Qinghai indigenous chickens; RJFs: red jungle fowls.

In total, 284 and 239 blood samples were collected from 5 low-altitude Sichuan chicken populations (Leshan, Chengdu, Dazhou, Yaan and Panzhihua) and 7 Tibetan chicken populations from Qinghai-Tibet Plateau localities (Diqing, Linzhi, Lhasa, Shannan, Haiyan, Aba and Ganzi), respectively (Fig 1). Genomic DNA was extracted from blood samples using phenol/chloroform following standard procedures [23]. The partial control region (approximately 500 bp) was amplified using the following previously published primers [2]: L16750: 5’-AGGACTACGGCTTGAAAAGC-3’ and H522: 5’-ATGTGCCTGACCGAGGAACCAG-3’. PCR amplification was performed in a 25-μL volume containing 1.25 μL of template genomic DNA, 12.5 μL of 2× Taq PCR Master Mix, 1.25 μL of each primer (10 pmol/μL), and 8.75 μL of ddH_2_O. The PCR conditions were as follows: 95°C for 3 min; followed by 33 cycles of 94°C for 35 s, 58.4°C for 35 s and 72°C for 1 min; and a final extension at 72°C for 10 min. All experimental procedures were approved by the Institutional Animal Care and Use Committee of the Institute of Animal Genetic and Breeding, Sichuan Agricultural University, Chengdu, China, under permit No. YCS-B20100804. Published control region sequences of chickens from localities in Xinjiang, Qinghai, Sichuan, Yunnan, Tibet and Red jungle fowls were downloaded from the database of National Center for Biotechnology Information (NCBI). The quality of sequences were checked according to the workflow described previously [24, 25]. Sequences with potential artificial recombination, surplus of mutations or phantom mutations were not used for further analysis. Taken together, we analyzed 1613 sequences in this study, including 1091 retrieved from NCBI and 522 sequences from our samples.

### Sequence analysis

The nucleotide diversity and haplotypes of all sequences were estimated with DnaSP 5.0 [26]. Network 5.0 software [27] was used to draw maximum parsimony median-joining (MP) network plots. All sequences were aligned with CLUSTAL W [28] prior to phylogenetic analysis using the Bayesian Evolutionary Analysis by Sampling Trees (BEAST) 1.7 [29]. The GTR substitution model with the invariant sites and gamma distribution (GTR+I+G) was selected as the best-fitting model using the jModelTest 2.1.1 [30]. The length of MCMC was set to 30,000,000 and sampled every 1,000 generations with 10% burn-in. The maximum clade credibility tree was created using TreeAnnotator [29]. DomeTree and MitotoolPy were used to identify mutations and standard haplogroups in each sequences [31].

## Results

### Genetic diversity of Tibetan and lowland fowls

In this study we obtained partial control region sequences of 522 individuals (S1 dataset), together with those published control region sequences from adjacent geographic regions, we generated a dataset containing 1613 sequences, including 276 Tibetan chickens, 461 Sichuan indigenous chickens, 743 Yunnan indigenous chickens, 14 indigenous chickens from Qinghai, 39 indigenous chickens from Xinjiang and 80 Red jungle fowls (RJFs). The overall information of Tibetan chickens as well as chickens from different populations were summarized in Table 1, within the Tibetan chickens from different populations, the number of polymorphic sites in each population varies from 17 in Linzhi to 27 in Haiyan, and the unique sequences from Aba, Diqing, Ganzi, Haiyan, Lhasa, Linzhi, Shannan and the unknown was 11, 9, 11, 15, 15, 5, 8 and 9, respectively. The highest haplotype diversity was found in Lhasa, while the average number of nucleotide differences of RJFs was greater than others. For those Tibetan chicken sequences retrieved from NCBI, the value of average number of nucleotide differences is higher than other Tibetan chicken populations, it means that those sequences are potentially obtained from different regions. Tajima's D test was not significant in all populations, indicating that neither balancing selection nor purifying selection occurred in all chicken populations. Taken together, a total of 42 haplotypes coming from 47 polymorphic sites were identified in Tibetan chickens, and the overall haplotype diversity, nucleotide diversity and average nucleotide differences were 0.925, 0.016 and 7.685, respectively. All of the genetic diversity parameters in Tibetan chickens did not have much differences from other lowland chickens of different localities, suggesting that the genetic diversity of Tibetan chickens is similar to the indigenous chickens of adjacent regions.

**Table 1:** Genetic diversity indices of Tibetan and lowland fowls.

	Population	Number of samples	Number of polymorphic sites	Number of haplotypes	Haplotype diversity	Nucleotide diversity	Average number of nucleotide differences	Tajima's D	Source
Tibetan fowls	Aba	36	21	11	0.86	0.011	5.144	0.054	Present study
	Diqing	25	21	9	0.76	0.011	5.183	-0.403	Present study
	Ganzi	23	24	11	0.862	0.013	6.150	-0.478	Present study
	Haiyan	60	27	15	0.884	0.013	6.362	0.317	Present study
	Lasa	49	33	15	0.904	0.014	6.636	-0.348	Present study
	Linzhi	13	17	5	0.808	0.014	6.872	1.074	Present study
	Shannan	33	21	8	0.642	0.008	3.864	-0.871	Present study
	Unknown	37	23	9	0.883	0.016	7.634	1.311	NCBI
	Total Tibetan chickens	276	47	42	0.925	0.016	7.685	-0.138	Present study + NCBI
Lowland fowls	Sichuan	461	47	51	0.893	0.014	6.613	-0.263	Present study + NCBI
	Yunnan	743	65	106	0.931	0.170	8.160	-0.416	NCBI
	Qinghai	14	19	11	0.967	0.012	6.604	0.430	NCBI
	Xinjiang	39	22	11	0.877	0.013	6.691	0.959	NCBI
	RJF	80	43	28	0.886	0.019	9.796	0.253	NCBI

Note: the geographical distribution of Tibetan chickens’ sequences downloaded from NCBI could not be retrieved, and therefore, their location is unknown.

### Phylogenetic relationships among Tibetan and indigenous chickens

**Fig 2:**
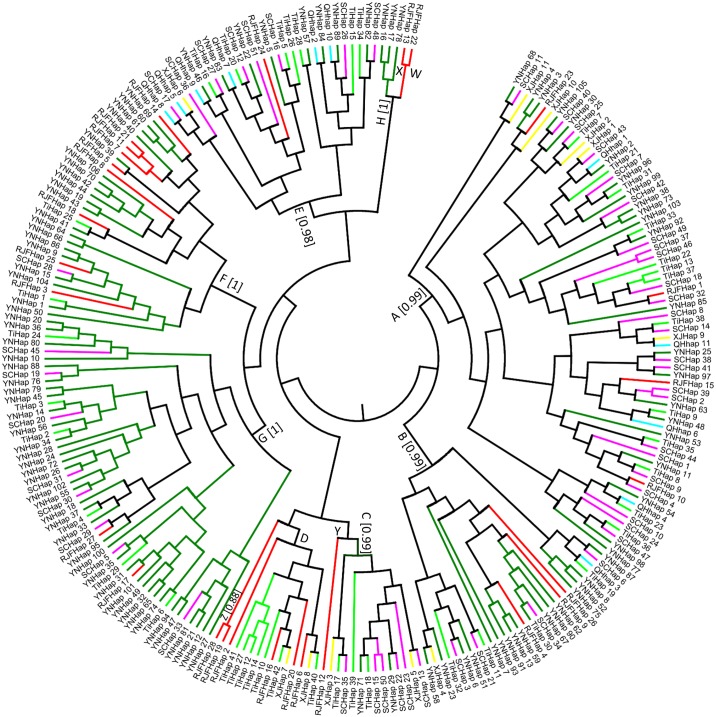
Phylogenetic tree of control region haplotypes constructed by Bayesian method. Values in parentheses are Bayesian posterior probability of haplogroups (if haplogroups with more than one individual) identified by Liu et al [17] and Miao et al [2]. Only Bayesian posterior probability of the clade higher than 0.5 are indicated above branches. Red lines: red jungle fowls (RJFs); dark green lines: Yunnan indigenous chickens (YN); pink lines: Sichuan indigenous chickens (SC); blue lines: Qinghai indigenous chickens (QH); yellow lines: Xinjiang indigenous chickens (XJ) and light green lines: Tibetan chickens (Ti).

To study the relationships among different populations, a phylogenetic tree of haplotypes was constructed by Bayesian method. Simultaneously, MitotoolPy was used to classify all the haplotypes as well as the sequences to different clades by diagnostic mutational motifs. As illustrated in Fig 2, the phylogenetic relationships generated by BEAST software is in agreement with the previous studies [2, 17]. Except for the paraphyletic haplogroup D, all haplogroups with more than one individual were supported by a robust posterior possibility value. Notably, like the neighbor-joining tree built of control region sequences, the RJFHap 6 which belongs to haplogroup C by diagnostic mutational motifs, was classified into clade D in our phylogenetic tree. As shown in Table 2 and Fig 1, clade A, B and G harbored haplotypes from different populations in this study, while no sequence from Xinjiang and Qinghai indigenous chickens was found in clade F and clade G. Clade D contained haplotype from Tibet Plateau, Xinjiang and RJFs, Clade H contained only indigenous chickens that were restricted to Yunnan, and only a few haplotypes represented a small number of RJFs belonging to clade W, X, Y and Z. As for Clade I, the rare south Asia haplotypes, was not found in those sequences. Those results clearly indicated that hybridization among Chinese chickens is extremely common.

**Table 2:** Geographical distribution of the major clades of different populations.

Population		Tibetan chickens	Sichuan	Yunnan	Qinghai	Xinjiang	RJFs
Clade A	Individual	66(23.913)	183(39.696)	202(27.187)	5(35.714)	15(38.462)	4(5.000)
	Haplotypes	13(30.952)	24(47.059)	22(20.755)	4(36.364)	5(45.455)	4(14.286)
Clade B	Individual	16(5.797)	44(9.544)	187(25.168)	2(14.286)	5(12.821)	22(27.500)
	Haplotypes	4(9.524)	4(7.843)	16(15.094)	1(9.091)	1(9.091)	3(10.714)
Clade C	Individual	9(3.261)	23(4.989)	12(1.615)	0	13(33.333)	18(22.500)
	Haplotypes	3(7.143)	6(11.765)	2(1.887)	0	2(18.182)	1(3.571)
Clade D	Individual	50(18.116)	0	0	0	2(5.128)	6(7.500)
	Haplotypes	7(16.667)	0	0	0	2(18.182)	3(10.714)
Clade E	Individual	73(26.449)	163(35.358)	45(6.057)	7(50.000)	4(10.256)	1(1.250)
	Haplotypes	7(16.667)	8(15.686)	8(7.547)	6(54.545)	1(9.091)	1(3.571)
Clade F	Individual	12(4.348)	2(0.434)	129(17.362)	0	0	20(25.000)
	Haplotypes	2(4.762)	1(1.961)	20(18.868)	0	0	9(32.143)
Clade G	Individual	50(18.116)	46(9.978)	157(21.131)	0	0	2(2.500)
	Haplotypes	6(14.286)	8(15.686)	35(33.019)	0	0	2(7.143)
Clade H	Individual	0	0	11(1.480)	0	0	0
	Haplotypes	0	0	3(2.830)	0	0	0
Clade W	Individual	0	0	0	0	0	1(1.250)
	Haplotypes	0	0	0	0	0	1(3.571)
Clade X	Individual	0	0	0	0	0	1(1.250)
	Haplotypes	0	0	0	0	0	1(3.571)
Clade Y	Individual	0	0	0	0	0	2(2.500)
	Haplotypes	0	0	0	0	0	1(3.571)
Clade Z	Individual	0	0	0	0	0	3(3.750)
	Haplotypes	0	0	0	0	0	2(7.143)

Note: numbers in parentheses indicate the proportion in this clade.

### Median-joining network profiles of Tibetan chickens in comparison to other populations

**Fig 3:**
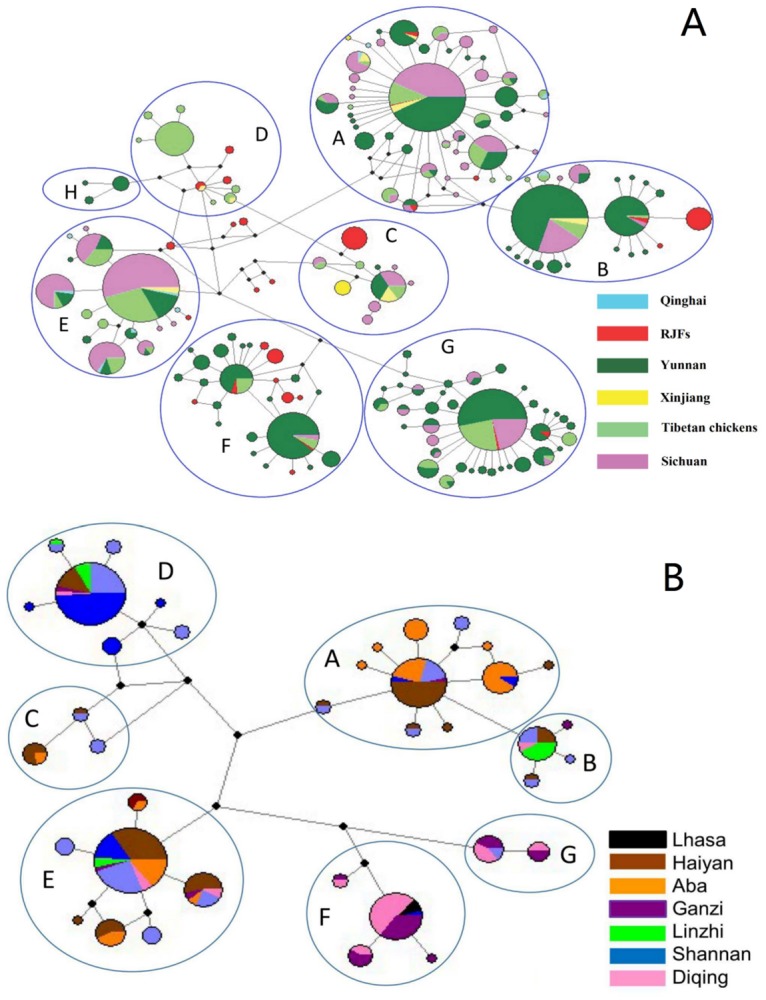
Maximum parsimony median-joining network of Tibetan chickens with (A) and without the chickens of adjacent regions (B). The domestic clades (A-H) are labeled. Node sizes are proportional to haplotype frequencies. The lines linking the nodes are proportional to the mutation steps. Black nodes indicate inferred steps not identified in the sampled populations. Colors within the circles represent chickens from different localities.

To investigate the relationships of haplotypes, we constructed network plot for Tibetan chickens with and without the chickens of adjacent regions (Fig 3A and 3B). The median-joining network revealed a complicated pattern among Tibetan chickens and other fowls (Fig 3A). Nevertheless, the majority of the haplogroups exhibited a star-like distribution profile, indicating a recent exponential population growth. In clade A, B, F and G, the core haplotypes occurred in Tibetan chickens, RJFs and indigenous chickens in adjacent provinces. As for clade C and E, sequences of RJFs were identified on the periphery of the plot; whereas, Tibetan chickens shared main haplotypes with other indigenous chickens. Importantly, only Tibetan chickens formed the core haplotype in clade D and this core haplotype differed at least by two mutation distance from other haplotypes. Fig 3B presented the network within Tibetan chickens sequenced in this study (the sequences of Tibetan chickens downloaded from NCBI may come from more than one population, and therefore, were not included in Fig 3B), and it was also consistent with a star like pattern. Seven clades (A-G) were found in Lhasa and the remaining Tibetan chickens clustered from three clades in Aba to six clades in Ganzi. It is noteworthy that, there were high frequency of samples from Diqing and Ganzi in clade F and G. However, those private haplotypes identified in clade D were, without expectation, almost distributed in all populations of Tibetan chickens (Fig 3B).

## Discussion

In contrast to slowly mutating genes, which provide data about ancient history, the control region of mitochondrial DNA exhibits high mutation rates and provides information about recent evolutionary history. To reconstruct the recent past, we used this marker to address the issue of the domestication event of Tibetan chickens. By extent the sample size, we, found for the first time, haplotypes not only belong to A, E, F and G [2, 17], but also dispersed in clade B, C and D. These results suggested that they are originated from different distinct maternal lineages.

Although Tibetan chickens have adapted to the extreme environment through specific genetic mechanisms [32, 33], our phylogenetic tree and network analysis indicated that they are not distinguishable from the indigenous chickens in surrounding areas. In addition, the frequencies and distribution of haplotypes of Tibetan chickens were similar to indigenous fowls in the adjacent regions. Furthermore, a total of seven haplogroups were found in Tibetan chickens which is higher than the indigenous population in each adjacent regions. Collectively, those evidences indicated that Tibetan chickens are most likely diverged from indigenous chickens in the surrounding areas of China rather than directly from red jungle fowls. In the history, two important trade routes namely Tangbo Ancient Road and Tea-horse Ancient Road linked the Qinghai-Tibet Plateau and the surrounding regions of China [34, 35]. Thus, as an easily carried species, chicken is probably introduced to Tibet Plateau from the northwest and southwest of China. However, the Qinghai-Tibet Plateau samples possess a great number of unique haplotypes (Fig 3A). There were a lot of haplotypes of Tibetan chickens presented in clade D and only a few shared with Xinjiang indigenous chickens. Our previous findings indicated that the distribution of clade D is associated with the dispersal of cockfighting cultures [17, 36], and indeed, Tibetan chickens appear more aggressive than other common chickens. Therefore, we propose that game fowls may have been introduced to Tibetan chickens. Nevertheless, it is hard to make conclusion about the origin of those haplotypes. Because almost all of these haplotypes shared an undiscovered mutation linkage at 355 and 391 sites [2] (S1 Table) and only a few samples (39 individuals) in Xinjiang have been sequenced. More importantly, we noticed that these private haplotypes are widely distributed in Tibetan chicken populations, suggesting that potential maternal lineages of game fowls have been introduced to Qinghai-Tibet Plateau for a long time.

As aforementioned, the 49 samples in Lhasa were assigned to seven domestic clades, because Lhasa is the economic center of Tibetan region, highest haplotype diversity and haplogroups distribution of fowls in this place may be caused by numerous trade activities. In contrast, some Tibetan populations possess specific haplotypes, for example, the results presented in Fig 3 indicated that the majority of samples of Diqing and Ganzi were classified into clade F and G. On the account of haplogroups F and G were mainly distributed in Yunnan and shared their core haplotypes with Tibetan chickens. We speculate that the origin of Tibetan chickens in Diqing and Ganzi can be traced back to Yunnan. However, we found that only a few individuals in Sichuan were distributed in Clade F and G. It is unlikely that the Sichuan act as a passageway for the gene flow from Diqing Tibetan chickens to Ganzi. Thus, we propose that the Tibetan chickens in Diqing are directly originated from Yunnan while a large number of individuals in Ganzi are introduced from the Diqing by breeding activities. In addition, sequences of clade F and G were absent in many places of Tibetan highland, indicating that and hybridization between Tibetan chickens and Yunnan indigenous fowls happened recently.

In summary, we sequenced and compared Tibetan chickens with different chicken populations in this study. The results indicated that the Tibetan chickens exhibit multiple origins, and they may originate from indigenous fowls in Yunnan, Sichuan, Xinjiang and/or game fowls. Additionally, breeding activities and hybridization may exist within or among different populations and the maternal lineages of Tibetan chickens is very complex. The obtained mtDNA phylogeny extends our understanding of the matrilineal history of chickens and more genetics or genomics studies need to be conducted in order to reveal the clear gene flow between or among different populations in the future.

## Supporting information

S1 Dataset Partial mtDNA control region sequences of 284 Sichuan indigenous chickens and 239 Tibetan chickens. (TXT)

S1 Table Summary of haplogroups and detail variants of each sequence used in this study. (XLSX)
